# Resistin-Like Molecule α Dysregulates Cardiac Bioenergetics in Neonatal Rat Cardiomyocytes

**DOI:** 10.3389/fcvm.2021.574708

**Published:** 2021-04-26

**Authors:** Bingdong Tao, Santosh Kumar, Jose Gomez-Arroyo, Chunling Fan, Ailan Zhang, John Skinner, Elizabeth Hunter, Kazuyo Yamaji-Kegan, Idris Samad, Alexander T. Hillel, Qing Lin, Wenqian Zhai, Wei Dong Gao, Roger A. Johns

**Affiliations:** ^1^Department of Anesthesiology and Critical Care Medicine, Johns Hopkins University, School of Medicine, Baltimore, MD, United States; ^2^Department of Anesthesiology, Shengjing Hospital, China Medical University, Shenyang, China; ^3^Department of Anesthesiology, Maryland University, School of Medicine, Baltimore, MD, United States; ^4^Department of Otolaryngology-Head and Neck Surgery, Johns Hopkins University, School of Medicine, Baltimore, MD, United States; ^5^Department of Anesthesiology, Tianjin Chest Hospital, Tianjin, China

**Keywords:** fatty acid oxidation, mitochondria, RELMα, heart failiure, oxygen consumption rate

## Abstract

Heart (right) failure is the most frequent cause of death in patients with pulmonary arterial hypertension. Although historically, increased right ventricular afterload has been considered the main contributor to right heart failure in such patients, recent evidence has suggested a potential role of load-independent factors. Here, we tested the hypothesis that resistin–like molecule α (RELMα), which has been implicated in the pathogenesis of vascular remodeling in pulmonary artery hypertension, also contributes to cardiac metabolic remodeling, leading to heart failure. Recombinant RELMα (rRELMα) was generated via a Tet-On expression system in the T-REx 293 cell line. Cultured neonatal rat cardiomyocytes were treated with purified rRELMα for 24 h at a dose of 50 nM. Treated cardiomyocytes exhibited decreased mRNA and protein expression of peroxisome proliferator-activated receptor gamma coactivator 1α (PGC-1α) and transcription factors PPARα and ERRα, which regulate mitochondrial fatty acid metabolism, whereas genes that encode for glycolysis-related proteins were significantly upregulated. Cardiomyocytes treated with rRELMα also exhibited a decreased basal respiration, maximal respiration, spare respiratory capacity, ATP-linked OCR, and increased glycolysis, as assessed with a microplate-based cellular respirometry apparatus. Transmission electron microscopy revealed abnormal mitochondrial ultrastructure in cardiomyocytes treated with rRELMα. Our data indicate that RELMα affects cardiac energy metabolism and mitochondrial structure, biogenesis, and function by downregulating the expression of the PGC-1α/PPARα/ERRα axis.

**Graphical Abstract:**
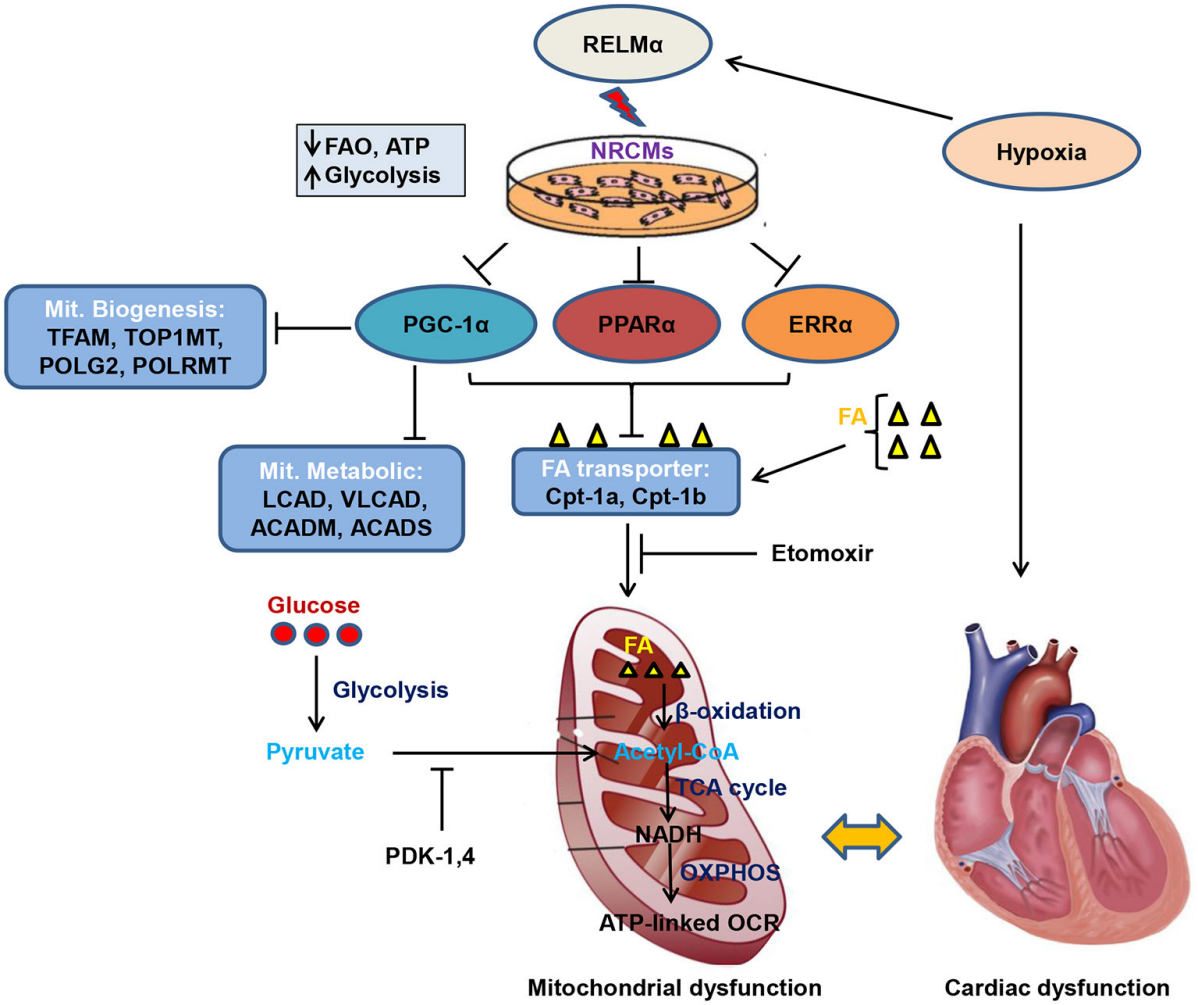
Schematic model of RELMα mediated mitochondrial and cardiac dysfunction. Rodent isoform of resistin RELMα, also known as hypoxia induced mitogenic factor (HIMF), attenuates adenosine triphosphate (ATP)-linked OCR, fatty acid oxidation (FAO) and partially increases glycolytic oxidation as compensatory mechanism in NRCMs. Mechanistically, RELMα attenuates PGC1α/PPARα/ERRα signaling axis which leads to down regulation of mitochondrial biogenesis genes (TFAM, TOP1MT, POLG2, and POLRMT), FAO metabolic genes (LCAD, VLCAD, ACADM, ACADS) as well as mitochondrial fatty acid (FA) transporter genes (Cpt-1a, Cpt-1b). Etomoxir (inhibitor of long chain FA) also limits FA availability to mitochondria, thus, less FAO (β-oxidation) further limits Acetyl-CoA production (substrate for TCA cycle). Slowdown of TCA cycle also impairs reduced NAD (nicotinamide adenine dinucleotide) i.e., NADH production utilized by oxidative phosphorylation (OXPHOS) pathway for ATP production through electron transport chain. RELMα partially increases glycolytic oxidation and increases pyruvate dehydrogenase kinase (PDK-1, 4) gene expression which also inhibits conversion of pyruvate to acetyl-CoA for TCA cycle. Persistent decrease in FAO and ATP-linked OCR leads to mitochondrial dysfunction which affects cardiac bioenergetics, thus, cardiac dysfunction.

## Highlights

- RELMα decreases mitochondrial fatty acid oxidation and increases glycolytic oxidation.
- RELMα affects cardiac bioenergetics by downregulating the PGC-1α/PPARα/ERRα axis.
- RELMα affects mitochondrial biogenesis, ultrastructure, and its function.
- RELMα decreases ATP -linked OCR by majorly limiting FA as a mitochondrial biofuel.
- RELMα participates in pulmonary arterial hypertension and right ventricular hypertrophy.

## Introduction

Pulmonary arterial hypertension (PAH) is characterized by a chronically increased right ventricular (RV) afterload, which frequently leads to RV failure and untimely death (1, 2). Although PAH-associated RV dysfunction has been classically explained as a consequence of pressure-overload, recent studies have demonstrated that the RV not only remodels differently between PAH types (3) but also adapts differently to a similar degree of biomechanical stress (i.e., high pulmonary pressures) (4, 5). In addition, experimental data have shown that isolated mechanical RV pressure-overload in rats subjected to pulmonary artery banding is insufficient to cause to RV failure (6), suggesting that load-independent factors might contribute to the transition from adaptive RV hypertrophy to RV failure (7).

In recent years, the idea that chronic inflammation plays a role in the pathogenesis and progression of PAH has gained momentum. Indeed, resistin-like molecule α (RELMα), also known as Found in Inflammatory Zone 1 (FIZZ1), or hypoxia-induced mitogenic factor (HIMF), belongs to a family of pro-inflammatory molecules that have been implicated in the development of PAH (8); however, the contributions of RELMα to RV failure are largely unknown. A consistent supply of energy is crucial for cardiac function. Cardiac ATP generation mainly relies on the conversion of fatty acids to energy via fatty acid oxidation in mitochondria (9). Decreased myocardial expression of the transcription factor peroxisome proliferator-activated receptor gamma coactivator 1-α (PGC-1α), a key factor in regulating mitochondrial biogenesis pathways, has been reported in experimental models of heart failure (10–12). Furthermore, it is significantly downregulated in the RVs of rats with severe PAH secondary to SU5416/hypoxia exposure, as well as in RV tissue from patients with severe PAH (13). However, this downregulation is not explained by pressure overload, hypoxia or SU5416 alone suggesting alternative mechanisms. In this study, we attempted to link RELMα to cardiac dysfunction by examining whether RELMα exerts direct cardiometabolic effects in addition to its effect on pulmonary vasculature. We show that RELMα inhibited fatty acid oxidation (FAO), ATP-linked OCR, mitochondrial biogenesis, thus, the slowdown of mitochondrial bioenergetics partly via downregulation of PGC-1α.

## Materials and Methods

### Recombinant RELMα Generation

Recombinant RELMα (rRELMα) protein was produced in T-REx 293 cells as previously described (11). Briefly, we integrated a pcDNA5/FRT/TO vector containing the mouse *Retnla* nucleotide sequence (NM_020509.3) into the genome of a Flp-In T-REx 293 cell line, in a Flp recombinase-dependent manner, using the Flp-In T-REx kit from Thermo Fisher (Waltham, MA). The transgene containing the *Retnla* sequence also included a tetracycline response element to allow for the production of rRELMα via a Tet-ON expression system. rRELMα was induced by adding tetracycline (1 μg/mL) to the T-REx 293 cells. rRELMα was purified from the REx 293 cell culture medium using the anti-FLAG M2 agarose affinity gel (Sigma, St. Louis, MO), a purified mouse IgG2B monoclonal antibody covalently bound to agarose. Purified rRELMα was finally extracted in PBS, which is also used for control treatment.

### Isolation, Culture, and Stimulation of Neonatal Rat Cardiomyocytes

Neonatal rat cardiomyocytes (NRCMs) were isolated from 1–3-day-old Sprague–Dawley pups as previously described (10). NRCMs were plated onto 6-well plates (5 × 10^5^ cells/mL, 2 mL per well) and cultured for 24 h in Dulbecco's modified Eagle medium (DMEM, Thermo Fisher) containing 10% fetal bovine serum (Gibco, Waltham, MA). NRCMs were serum-starved in DMEM containing 0.1% insulin transferrin selenium (Thermo Fisher) for 24 h and then incubated with rRELMα at a final concentration of 50 nM for 24 h (the number of NRCMs changed minimally during this time). The dose of 50 nM was selected for downstream experiments based on a dose-response curve (Supplementary Figure 1A). RELMα (10–100 nM) stimulation increases cell size in dose dependent manner (Supplementary Figure 1B). After stimulation, cells were harvested for quantification of total mRNA and protein.

### Quantitative PCR

Total RNA was isolated with the RNeasy Mini Kit (#74104, Qiagen) according to the manufacturer's protocol. Total RNA (500 ng) was reverse transcribed into cDNA. Quantitative PCR was carried out on an ABI 7500 fast real-time PCR system (Applied Biosystems, Foster City, CA). Fold changes in gene expression were acquired by using the delta method and normalization to 18S rRNA. The gene specific primers used in this study are listed in Supplementary Table 1.

### Western Blot Analysis

Cold RIPA buffer (50 mM Tris pH 8.0, 1% non-idet P40, 0.5% deoxycholate, 0.1% SDS, 150 mM NaCl) supplemented with 10 μL/mL phosphatase inhibitor cocktail 3 and cocktail 2 (Sigma, St. Louis, MO) was used to harvest the total protein extract. Protein concentration was determined by using Pierce^TM^ BCA protein assay kit (# 23227, Thermo Scientific) based on absorbance assay. 20 μg of total protein extract from indicated experimental condition was separated by sodium dodecyl sulfate-polyacrylamide gel electrophoresis (Bio-Rad, Hercules, CA) and transferred to polyvinylidene difluoride membranes (Bio-Rad). Non-specific binding was blocked in non-fat milk (5%) before the membranes were incubated with specific primary antibodies at 4°C overnight followed by appropriate secondary antibodies for 1 h at room temperature. Primary antibodies used included PGC-1α (1:1000), PPARα (1:1000), ERRα (1:1000), ACADM (1:1000), and GAPDH (1:3000), all from Sigma. Goat-anti-rabbit and goat-anti-mouse secondary antibodies were purchased from Bio-Rad and donkey-anti-goat secondary antibody was purchased from R&D (Minneapolis, MN). Signals were detected by enhanced chemiluminescence (GE Healthcare, Buckinghamshire, UK) and exposure to on X-ray film. Films from at least three individual experiments were scanned and densitometric analysis was performed with ImageJ software.

### *In vitro* Determination of Oxygen Consumption Rates

Oxygen consumption rate (OCR) and extracellular acidification rate (ECAR) of intact NRCMs were measured simultaneously on an XF24 Extracellular Flux Analyzer (Seahorse Agilent Technologies, North Billerica, MA) (14) using cell mito stress test kit (#103015-100, Seahorse Agilent Technologies). NRCMs were seeded in XF24 V7 cell culture microplates at a density of 5 × 10^5^ cells/well/mL and treated as described previously. First, NRCMs were gently washed twice with fatty acid oxidation assay medium and incubated for 1 h at 37°C without CO_2_, according to the manufacturer's protocol. To test the oxidation of fatty acids, we used palmitate-bovine serum albumin (0.16 mM) as a substrate (#102720-100, Seahorse Technologies). To investigate the mitochondrial respiratory capacity, we injected oligomycin (1 μM, ATP-synthase inhibitor), FCCP (2 μM, electron transfer chain accelerator), and a combination of rotenone (0.5 μM, Complex I inhibitor) and antimycin A (0.5 μM, Complex III inhibitor) into the medium at 34, 58, and 85 min, respectively. After the assay, OCR based basal respiration, maximal respiration, ATP-linked OCR, and proton leak were measured.

To further asses the direct role of long chain fatty acid oxidation as a major biofuel in the altered mitochondrial metabolism under RELMα stimulation, we performed above mentioned cell mito stress in presence of specific inhibitor of long chain fatty acid to mitochondria, Etomoxir, (#103260-100, Seahorse Agilent Technologies) on XF96 extracellular Flux analyzer according to manufacturer's protocol (15). NRCMs were seeded in XF96 cell culture microplates (#101085-004, Seahorse Agilent Technologies) at a density of 4 × 10^4^ cells/well/100 μL and treated with RELMα as described previously. Etomoxir was specifically pretreated 15 min before the assay while other regent's treatments timing were similar to above-mentioned cell mito stress test. From each XFp cell culture microplate, amount of protein at least from triplicates of control and NRVMs exposed to RELMα with or without Etomoxir were measured using Pierce^TM^ BCA protein assay kit (# 23227, Thermo Scientific). Data were analyzed with Wave Desktop 2.6.1 software and a Microsoft Excel Macro provided by Agilent Technologies. Final results were expressed after normalizing the equal amount of protein for each experimental condition.

### Transmission Electron Microscopy

Transmission electron microscopy (TEM) was performed as described previously (16). Briefly, cardiomyocytes samples were fixed in 2.5% glutaraldehyde and 3 mM MgCl_2_ in 0.1 M sodium cacodylate buffer (pH 7.2) for 1 h at room temperature, and then were post-fixed in 1% osmium tetroxide in buffer for 1 h on ice in the dark, rinsed in 0.1 M maleate buffer, and then stained for 1 h in the dark with 2% uranyl acetate (0.22 μm filtered) in 0.1 M maleate buffer. After the staining procedure, samples were dehydrated in a graded series of ethanol and embedded in Eponate 12 (Ted Pella, Redding, CA) resin. Samples were polymerized at 37°C for 2–3 days before being transferred to 60°C overnight.

Regions of interest were identified under a standard phase microscope, and 3-mm discz were removed and mounted onto blank EPON blocks for sectioning. Thin sections, 60–90 nm, were cut with a diamond knife on the Reichert-Jung Ultracut E ultramicrotome and placed onto 2 × 1 mm copper slot grids. Grids were stained with 2% uranyl acetate in 50% methanol followed by lead citrate at 4°C. Samples were observed with a Philips CM120 transmission electron microscope at 80 kV, and images were captured with an AMT CCD 2,080 × 2,048 pixel, side-mount AMT XR80, high-resolution, high-speed camera. Ten images from different fields of each sample were captured at random. An investigator blind to the treatment group evaluated the density, distribution, size, and shape of the mitochondria using ImageJ software (NIH).

### Mitochondrial DNA and Nuclear DNA Copy Number

Primary cultured NRCMs were serum starved for overnight followed by treatment with RELMα for 24 h. Total DNA was isolated using gDNA kit (#10223, Qiagen Genomic-tip 20/G). To determine the mitochondrial DNA (mit-DNA) copy number in the samples, two separate quantitative PCR amplifications (Applied Biosystem) were carried out by using two different set of primers (Supplementary Table 1). First one is used to amplify part of the mitochondrial-DNA D-loop region, and the second one is used for nuclear hexokinase-2 gene. Quantitative PCR reactions of 20 μL contained 1× Power SYBR Green PCR master mix (Thermo Fisher Scientific), 0.5 μM forward primer, 0.5 μM reverse primer and 5 ng of DNA. The PCR program consisted of an enzyme activation step of 10 min at 95°C, followed by 40 cycles of a denaturing step of 15 s at 95°C, an annealing and elongation step of 1 min at 60°C, and a reading of the fluorescence. The mit-DNA copy number was calculated with the formula: 2 × 2–^(CtmitDNA−CtnDNA)^, where Ct mit-DNA is the cycle threshold for mt-DNA and Ct n-DNA is the cycle threshold for nuclear DNA.

### Animals and Hypoxia-Induced Pulmonary Hypertension (PH) Model

Age-matched 8–12-weeks and 150–200 g male Sprague-Dawley rats (Charles River Laboratories) were used for all hypoxia experiments. Given reports (17) that estrogens exhibit protective effects in classical rodent chronic hypoxia-induced PH models, we used only male rats to produce severe hypertensive symptoms. Animals were maintained on a 12/12-h light/dark cycle with access to normal laboratory diet (Teklad global 18% protein rodent diet; Envigo) and chlorinated water *ad libitum*. Cage bedding was also from Envigo (7097 Teklad corncob bedding). Animal housing and experimental protocols were approved by the Animal Care and Use Committee of Johns Hopkins University. For induction of the chronic hypoxia-induced PH model, rats were exposed to 10.0% O_**2**_ (hypoxia) for 21 days and then sacrificed and processed as per experimental protocol. Control rats were exposed to normal room air (20.8% O_**2**_, normoxia). After day 21, rats were killed and processed for histological examination. Heart, lung and other tissues were stored at −80°C till further use. We ensured that experiments were unbiased by following the recent guidelines for PH preclinical and translational research (18). Animals were randomized to each group, and group sizes were determined by power calculations. Investigators who assessed the imaging, hemodynamics, and histological outcomes in all PH animal models were blinded to group assignment.

### Colorimetric Bromodeoxyuridine (BrdU) Assay

A colorimetric non-radioactive reagent bromodeoxyuridine (BrdU) was used to measure proliferation of NRCMs, as described in manufacturer's protocol (Roche/Sigma-Aldrich). In brief, cardiomyocytes were seeded in 96-well plates (1 × 10^4^ cells/100 μL/well) and allowed to grow in 10% FBS, starved in serum-free medium for 24 h, followed by treatment with PBS or rRELMα for 24 h. Subsequently, Cardiomyocytes were labeled with 10 μM BrdU and re-incubated for another 16–24 h, followed by fixation (30 min, 20–25°C) and incubation with HRP-coupled anti-BrdU-antibody (90 min, 20–25°C). Thereafter, 100 μL substrate solution (tetramethyl-benzidine) per well was added, and the plate was incubated for 5–30 min at room temperature until blue color development was sufficient. Immediately, absorbance was measured at 370 nm (reference wavelength, 492 nm) in an ELISA plate reader. The cardiomyocytes viability was defined as the relative absorbance of treated vs. untreated control cells.

### Cell Surface Area Measurement

Primary cultured NRCMs were serum starved for overnight followed by PBS or RELMα (50 nM) stimulation. Real-time live cell images were captured using the IncuCyte S3 Live-Cell Analysis System (Essen BioScience, Inc., Ann Arbor, MI), courtesy of the Anesthesiology and Critical Care Medicine Functional Imaging Core of Johns Hopkins University School of Medicine, USA. Analysis was carried out using the IncuCyte software to calculate cardiomyocytes area. The cell surface area was quantified by measuring cardiomyocytes from 16 different randomized microscopic fields in each group (under a 20X objective), and the mean cell surface area normalized to the untreated control group were calculated.

### Statistical Analysis

Three wells per group were used in each cell culture experiment, and at least three cell culture experiments were repeated in this study. Data are expressed as mean ± SEM. Statistical analyses between two groups were performed by unpaired Student's *t*-test. Differences between multiple groups were tested by one-way ANOVA and levels of significance were determined using Bonferroni's multiple comparisons test (Graphpad Prism 6 Software Inc., San Diego, CA). *p* < 0.05 was considered statistically significant.

## Results

### rRELMα Significantly Downregulates Fatty Acid Oxidation-Related Genes in NRCMs

**Figure 1:**
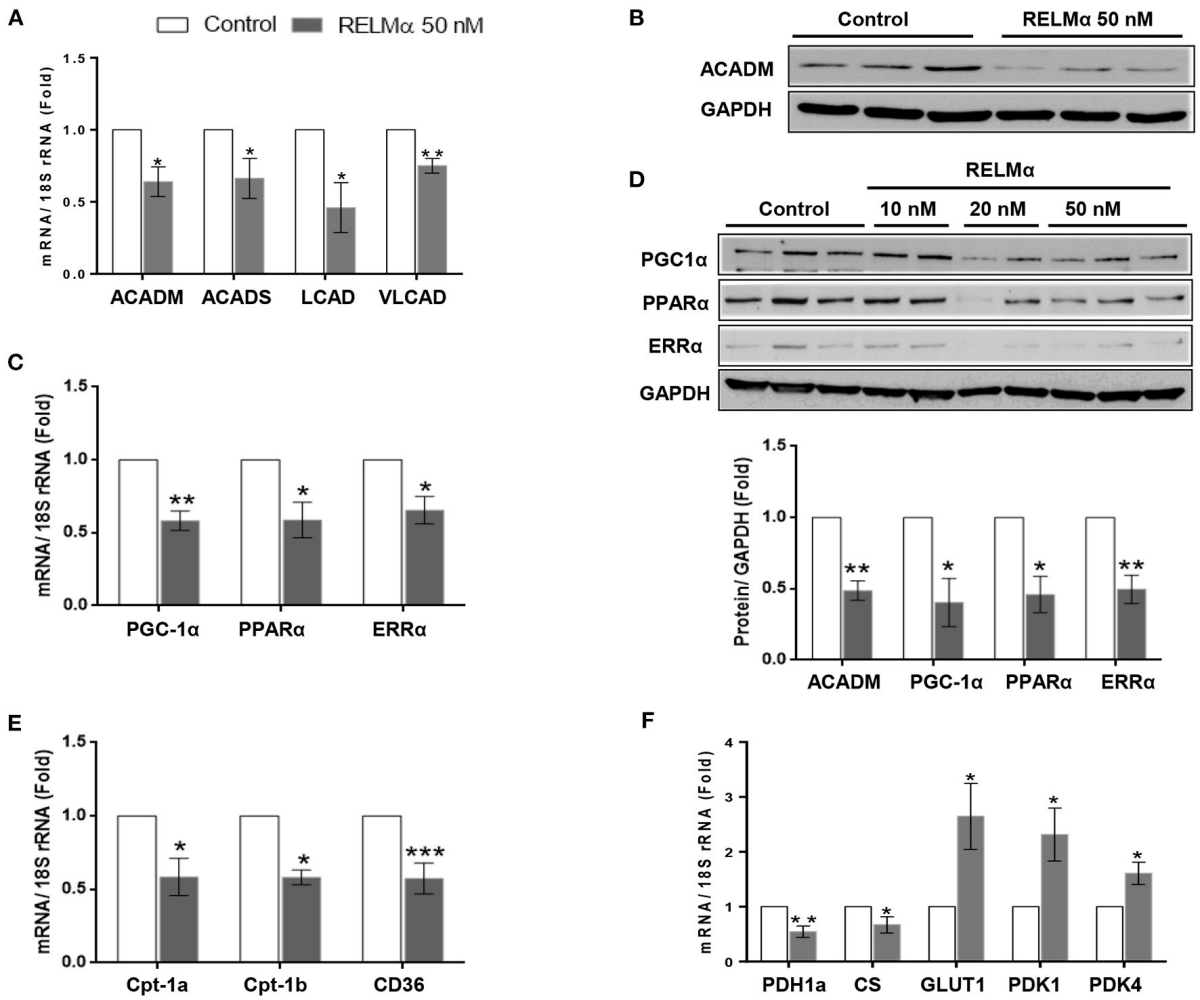
RELMα downregulates genes related to cardiac energy metabolism. **(A)** Quantitative PCR analyses of acyl-coenzyme A dehydrogenase straight chain (ACADM), acyl-coenzyme A dehydrogenase short chain (ACADS), long-chain acyl-CoA dehydrogenase (LCAD), and very long-chain acyl-CoA dehydrogenase (VLCAD) in total mRNA derived from neonatal rat cardiomyocytes (NRCMs) exposed to RELMα (50 nM). **(B)** Immunoblotting of ACADM, peroxisome proliferator-activated receptor gamma coactivator 1-α (PGC-1α) and transcription factors PPARα and ERRα in total protein extract derived from NRCMs exposed to RELMα at indicated concentration. **(C)** Quantitative PCR analyses of peroxisome PGC-1α, PPARα and ERRα in mRNA derived from NRCMs exposed to RELMα (50 nM). **(D)** Relative quantification for immunoblots of ACADM, PGC-1α, PPARα, and ERRα in total protein extract isolated from NRCMs exposed to RELMα (50 nM). **(E)** Quantitative PCR analyses of carnitine palmitoyltransferase-1a and 1b (CPT-1a and CPT-1b) and CD36 in total mRNA derived from NRCMs exposed to RELMα (50 nM). **(F)** Quantitative PCR analyses of pyruvate dehydrogenase 1a (PDH1a), citrate synthase (CS), glucose transporter 1 (GLUT1), pyruvate dehydrogenase kinase 1, and 4 (PDK1 and PDK4) in total mRNA derived from NRCMs exposed to RELMα (50 nM). *n* = 3–4/group. *, **, ***p < 0.05, 0.01, 0.001 vs. control group.

To test whether RELMα directly alters the expression of key enzymes involved in fatty acid oxidation (FAO), we exposed primary cultured NRCMs to rRELMα. We found significant decrease in C-4 to C-12 straight chain of acyl-coA dehydrogenases (ACADM) and C-2 to C-3 short chain of acyl-coA dehydrogenases (ACADS) gene involved in fatty acid oxidation. Similarly, we also found significant decrease in other biomarkers of fatty acid oxidation like long-chain acyl-CoA dehydrogenase (LCAD), and very long-chain acyl-CoA dehydrogenase (VLCAD) at transcript level as compared to control (Figure 1A). Furthermore, we also found that straight chain of acyl-coA dehydrogenases (ACADM) were down regulated at the protein levels (Figure 1B).

### rRELMα Downregulates the Expression of PGC-1α, PPAR-α, ERRα, and Mitochondrial Metabolic Genes in Isolated NRCMs

In non-cardiac cell lines, the cytokine resistin, a human homolog of RELMα, impairs mitochondrial function via downregulation of PGC-1α (19). Because the RV tissue from rats and humans with severe PAH is characterized by downregulation of PGC-1α, as well as multiple downstream fatty acid oxidation genes, we hypothesized that RELMα could affect cellular metabolism via downregulation of PGC-1α. Indeed, cultured NRCMs stimulated with rRELMα showed approximately a 45% reduction in expression of PGC-1α and its corresponding transcription factors PPARα and ERRα at the transcript (Figure 1C) and protein levels (Figure 1D). Since GAPDH is a glycolytic enzyme, we also performed immunoblotting of cytoskeletal protein beta-Tubulin as loading control and found similar expression as control with RELMα (50 nM) stimulation (Supplementary Figure 4), which suggest no conflict with proposed mitochondrial bioenergetics study. Previous studies suggest that PGC-1α regulates cardiac metabolism by modulating the expression of multiple genes required for mitochondrial metabolism and biogenesis, including the aforementioned *Acadm* and *Acads* (20), along with other genes required for fatty acid transport into the cell like *Cd36* (21) and mitochondrial transporter gene carnitine palmitoyltransferase-1A, *Cpt-1a* (13), and, its cardiomyocytes specific isoform *Cpt-1b* (22). In agreement, our data indicate that RELMα also downregulated the gene expression of major lipid transporters *Cpt-1a, Cpt-1b, and CD36* in NRCMs (Figure 1E).

In order to establish partial *in vivo* support of previous *in vitro* findings, we used hypoxia-induced PH and RV hypertrophy rat model. Expectedly, we found increased pulmonary arterial vessel wall thickness (Supplementary Figures 2A,B) and RV hypertrophy features like right ventricular systolic pressure (RVSP), Fulton's index [Right Ventricle weight/ Left Ventricle + Septum weight], and hypertrophic genes expression (ANP, BNP, and β-MHC) as shown in Supplementary Figures 2C–E, respectively. In agreement with cellular model, we also found decreased expression of cardiomyocytes specific genes related to FAO (LCAD, VLCAD, Cpt-1b) in hypoxic RV tissue (Supplementary Figure 2F). Interestingly, we found increased RELMα mRNA (Supplementary Figure 2F) and its corresponding protein (Supplementary Figure 2G) level expression in hypoxic RV tissue. Taken together, our data partially highlight an important role of RELMα in altered mitochondrial metabolic FAO genes in cellular as well as animal model.

Whereas, FAO accounts for 60–90% of the energy needed for a healthy heart, glucose oxidation accounts for approximately 10–40% (23). Because the metabolic remodeling gene profile seen during RV failure in experimental models (13) is characterized not only by a change in FAO but also by changes in glucose oxidation, we measured the expression of genes encoding pyruvate dehydrogenase and citrate synthase, two critical proteins for mitochondrial glucose metabolism. Figure 1F (left panel) shows that RELMα led to a downregulation of genes for both pyruvate dehydrogenase 1 (*Pdh1*a) and citrate synthase (*CS*). Interestingly, the glycolysis-related genes, glucose transporter 1 (*Glut1*), a glucose co-transporter, and pyruvate dehydrogenase kinase 1 and 4 (*Pdk1, and Pdk4*), the enzyme that inactivates pyruvate dehydrogenase and inhibits the conversion of pyruvate to acetyl-CoA for the TCA (tricarboxylic acid) or Krebs cycle, were significantly upregulated compared to levels in the non-stimulated control group (Figure 1F-right panel). Taken together, our data suggest that RELMα may directly affect oxidative metabolism, while upregulating glycolysis in NRCMs, partly via downregulation of PGC-1α, the master co-activator of cardiac metabolism.

### rRELMα Decreases Mitochondrial ATP-Linked Oxidative Metabolism While Increasing Glycolysis in Isolated NRMCs

**Figure 2:**
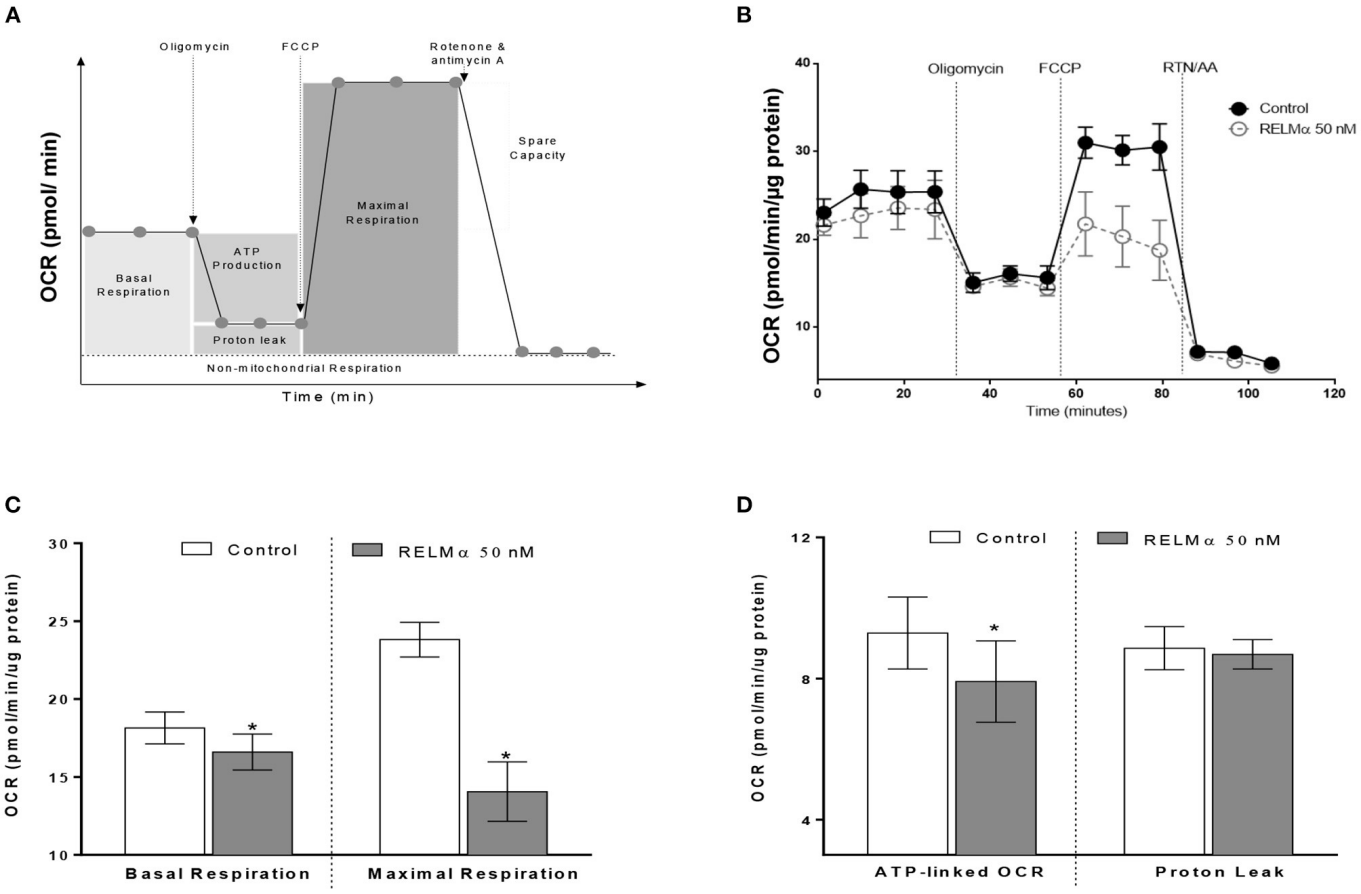
Neonatal rat cardiomyoctyes (NRCMs) challenged with RELMα exhibit a decreased oxygen consumption rate (OCR). **(A)** Schematic diagram of OCR kinetics. **(B)** OCR profile in control and RELMα-treated cardiomyocytes. After four baseline measurements, oligomycin (1 μM, ATP-synthase inhibitor), FCCP (2 μM, electron transfer chain accelerator), and a combination of rotenone (0.5 μM, Complex I inhibitor) and antimycin A (0.5 μM, Complex III inhibitor) were injected into the NRCMs at the indicated times for assessment of basal mitochondrial respiration, ATP-linked OCR, proton leak, maximal respiration, and reserve capacity. **(C)** OCR based basal and maximal respiration in control and RELMα-treated cardiomyocytes. **(D)** OCR based ATP-linked OCR and proton leak measurement in cultured cardiomyocytes. *n* = 5 per group. *p < 0.05 vs. control group.

To further evaluate whether the RELMα-induced down-regulation of mitochondrial oxidative metabolism–related genes actually translated into abnormal cellular metabolism, we measured the OCR and ECAR in NRCMs with the XF24 Extracellular Flux Analyzer. Briefly, NRCMs were provided with palmitate-BSA as a substrate for fatty acid oxidation and OCR was assessed at multiple time points before and after the addition of different uncouplers (Figure 2A) of the mitochondrial electron transport chain (ETC). These uncouplers allow for the evaluation of OCR based basal and maximal respiration, ATP-linked OCR, and non-mitochondrial respiration. As shown in Figures 2B,C, NRCMs exposed to RELMα (24 h prior to evaluation), exhibited a decreased basal and maximal OCR as well as SRC compared to unstimulated control group. To evaluate whether the change in basal OCR was due to a decrease in ATP-linked mitochondrial OCR or non-ATP linked OCR (secondary to electron transport chain proton leak), oligomycin (an ATP-synthase inhibitor) was automatically added to the microplates after three independent OCR readouts. The decrease in basal OCR mainly dependent on a change in ATP-linked OCR, as the proton leak was not significantly different between cells treated with RELMα and unstimulated controls (Figure 2D). After recording oligomycin-insensitive OCR, FCCP (electron transfer chain accelerator) was injected and OCR was recorded. FCCP targets the inner mitochondrial membrane, mimicking increased energy demand, and allows for evaluation of the maximal respiration capacity that cells have to reduce oxygen into water, as part of normal respiration.

**Figure 3:**
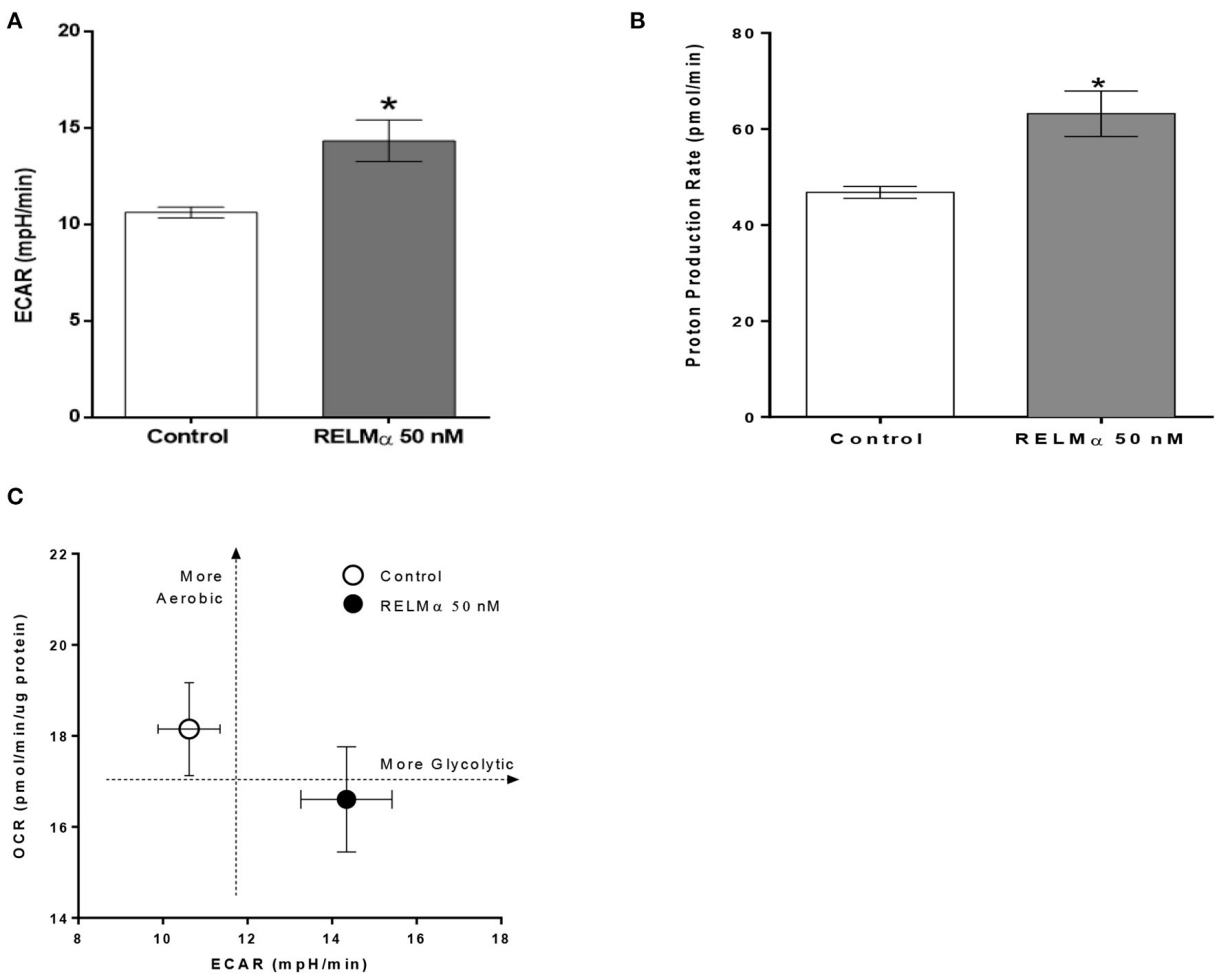
Neonatal rat cardiomyoctyes (NRCMs) challenged with RELMα exhibit an increased ability to augment glycolysis. **(A)** Extracellular acidification rate (ECAR), **(B)** Proton production rate, and **(C)** Metabolic profile of the stimulatory effect of RELMα on oxygen consumption rate (OCR) and glycolysis in primary cultured cardiomyocytes. The OCR (y-axis) and ECAR (x-axis) were plotted against one another at the same time. *n* = 5 per group. *p < 0.05 vs. control group.

As shown previously (Figures 2B,C) treatment with RELMα significantly decreased basal and maximal respiration of NRCMs. In addition to oxidative metabolism, glycolysis also contributes to a small fraction of the energy production. Therefore, when oxidative metabolism is reduced, the cellular glycolytic rates increase to compensate for a decrease in mitochondrial energy production. To determine whether glycolysis was altered after RELMα exposure, we evaluated the production of lactate in the cell medium (in terms of ECAR) and derived the proton production rate using the XF24 Analyzer. Both ECAR (Figure 3A) and proton production rate (Figure 3B) were greater in NRCMs treated with RELMα than in the vehicle treated control group. When OCR and ECAR were plotted against one another (Figure 3C), rRELMα-treated myocytes exhibited decreased oxidative metabolism and increased anaerobic glycolytic metabolism. Taken together, these data strongly suggest that RELMα significantly affects mitochondrial metabolic parameters (decreased basal and maximal respiration), signaling pathways (decreased FAO, and, increased glycolytic oxidation), and, bioenergetics (decreased ATP-linked OCR, increased ECAR and proton production) in the ETC of cardiomyocytes.

### rRELMα Limits FA Biofuel Required for Mitochondria Bioenergetics in NRMCs

**Figure 4:**
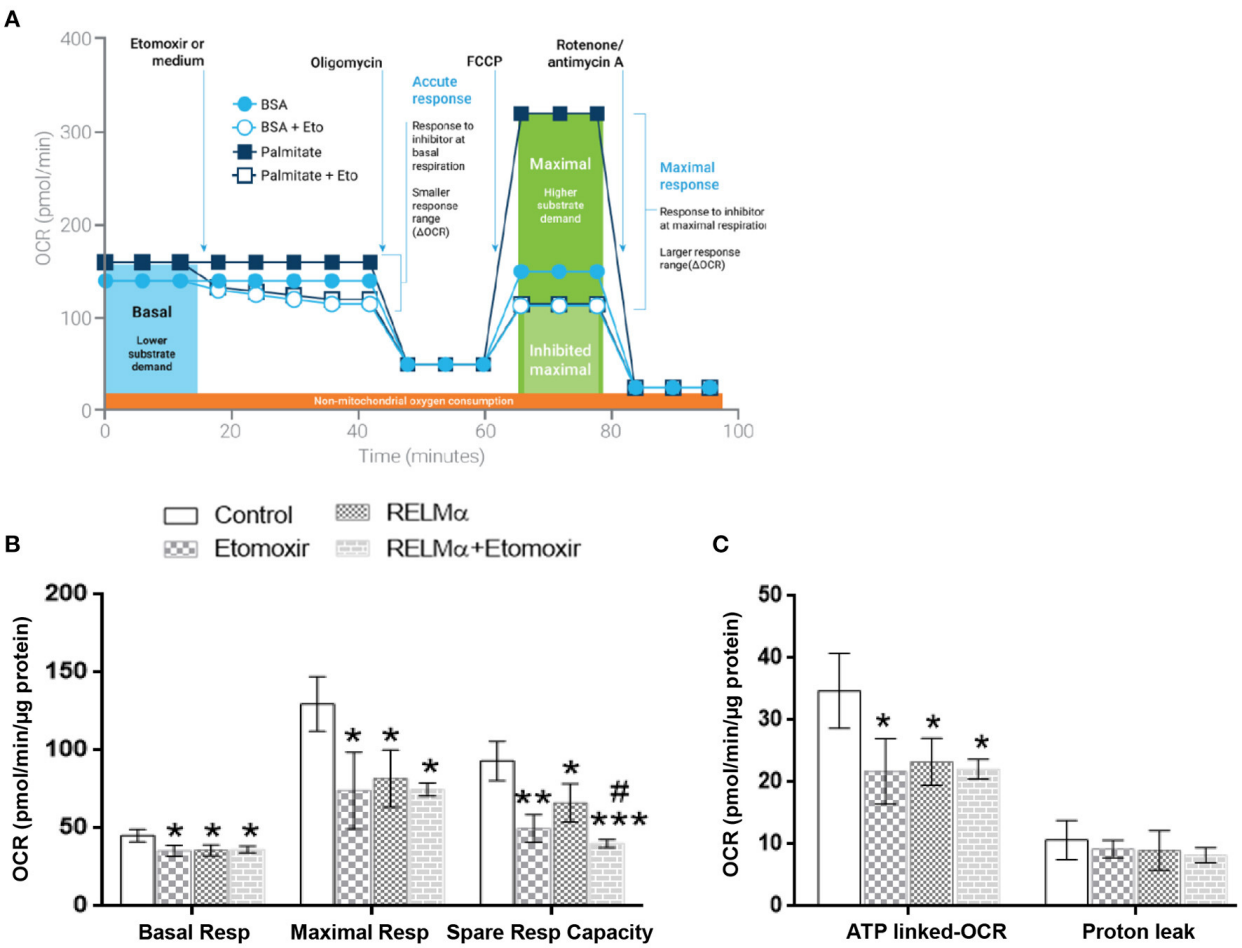
Neonatal rat cardiomyocytes (NRCMs) challenged with RELMα or Etomoxir alone or in combination exhibit decreased OCR based mitochondrial bioenergetics. **(A)** Schematic representation of fatty acid oxidation assay with Etomoxir (courtesy Agilent Technologies) in presence of Palmitate-BSA as substrate to primary cultured cardiomyocytes. Cardiomyocytes were treated with either assay media and/or Etomoxir (final concentration 4 μM,) as port A injection. After three baseline measurements, NRCMs were subjected to a final concentration of oligomycin (1 μM), FCCP (2 μM), and a combination of rotenone (0.5 μM) and antimycin A (0.5 μM) as ports B, C, and D injections, respectively, at the indicated times for assessment of basal mitochondrial respiration, maximal respiration, spare respiratory capacity, ATP-linked OCR, and proton leak. **(B)** Basal respiration, maximal respiration and spare respiratory capacity in cardiomyocytes treated with RELMα with or without Etomoxir. **(C)** OCR based ATP-linked OCR and proton leak in cardiomyocytes treated with RELMα with or without Etomoxir. *n* = 3 per group. *, **, ***p < 0.05, 0.01, 0.001 vs. control group. ^#^*p* < 0.05 vs. RELMα group.

**Figure 5:**
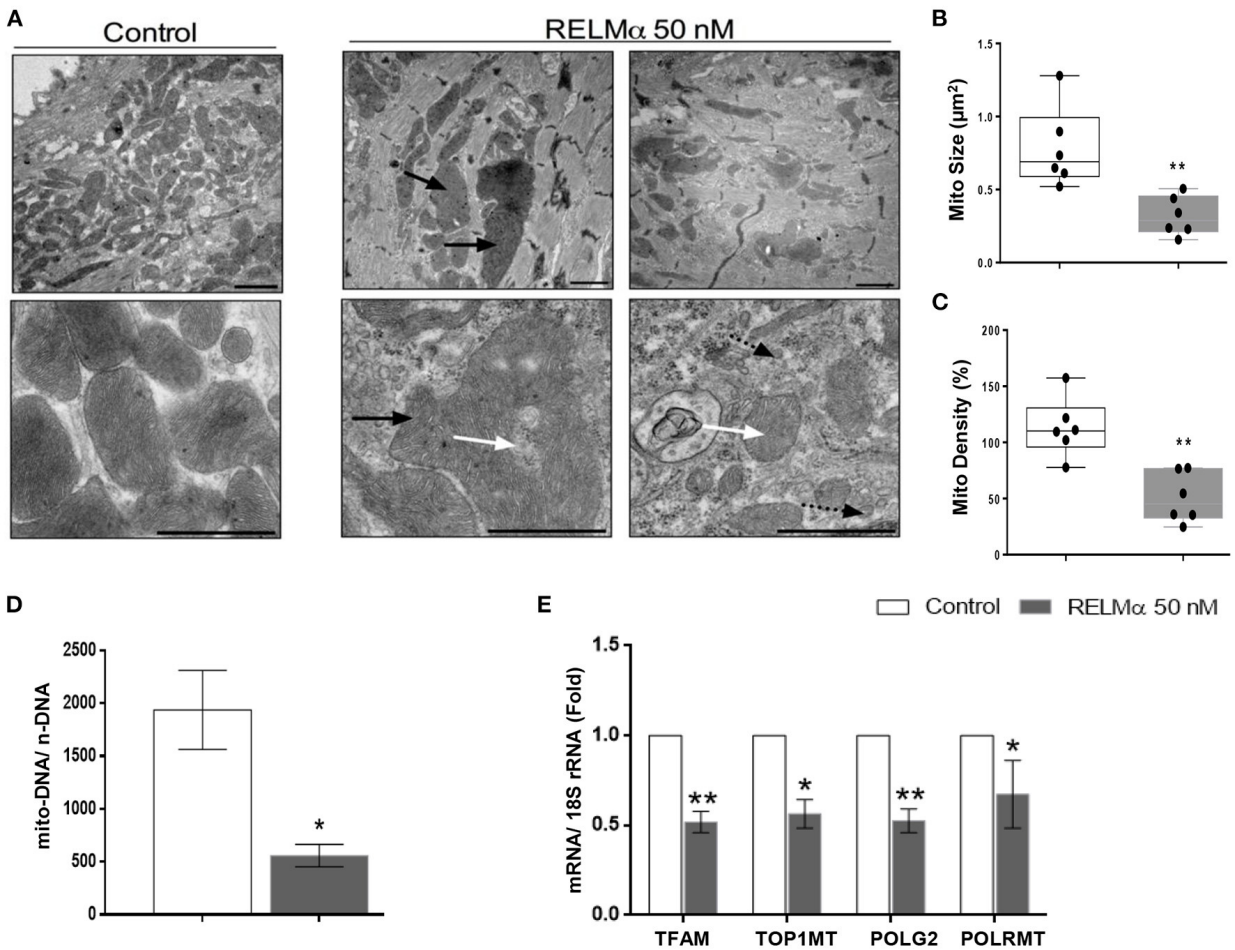
RELMα impairs mitochondrial ultrastructure, biogenesis and its replication machinery in NRCMs. **(A)** Representative electron micrographs taken from NRCMs stimulated with or without recombinant RELMα. Low magnification images (upper) were used to evaluate mitochondrial density, and high magnification images (lower) were used to investigate the ultrastructure of mitochondria. Arrows indicate structurally abnormal mitochondria (black arrow: giant mitochondria, white arrow: abnormal cristae structure, black arrow with dotted tail: small, fragmented mitochondria). Scale bars are 1 μm. **(B)** Representative electron micrographs showing mitochondrial morphology quantitated as average mitochondrial size (μm^**2**^), and **(C)** mitochondrial density per microscopic field by using imageJ software. **(D)** Estimation of mitochondrial DNA copy number (mito-DNA/nuclear DNA ratio) by quantitative PCR in NRCMs. **(E)** Quantitative PCR analyses of mitochondrial transcription factor A (TFAM), mitochondrial topoisomerase I (TOP1MT), mitochondrial DNA polymerase subunit gamma 2 (POLG2), and mitochondrial DNA-directed RNA polymerase (POLRMT) in NRCMs. *n* = 4–7 per group. *p < 0.05, **p < 0.01 vs. control group.

In order to assess the direct role of long chain fatty acid (FA) as a major mitochondrial biofuel under RELMα stimulation, we measured aforementioned OCR-based mitochondrial metabolic parameters in presence or absence of Etomoxir (specific inhibitor of long chain fatty acid import in to mitochondrial). Schematic representation of OCR-based kinetics (courtesy Agilent Technologies) for the FAO assay in presence of XF-palmitate-BSA as substrate with or without Etomoxir (Figure 4A). In agreement with model figure, our data also suggest there is a significant decrease in OCR based basal and maximal cellular respiration, as well as SRC with RELMα and Etomoxir stimulation alone (Figure 4B). However, we did not find any co-stimulatory effects of RELMα and Etomoxir on basal and maximal cellular respiration in cardiomyocytes (Figure 5B, left and middle panel). Interestingly, the SRC was further decreased upon co-stimulation with RELMα and Etomoxir stimulation in cardiomyocytes (Figure 5B, right panel), clearly indicates major contribution of long chain fatty acid as mitochondrial biofuel. Furthermore, we also found significant decrease in ATP-linked OCR upon RELMα and Etomoxir stimulation alone (Figure 4C, left panel). However, unlike SRC, it was not further decreased upon their co-stimulation. We did not find any significant difference in proton leak upon RELMα and Etomoxir stimulation alone or with their co-stimulation as compared to control (Figure 4C, right panel). Concurrent to previous cardio-metabolic findings, these data highlights novel dependency on long chain fatty acid as major mitochondrial biofuel for maintaining cardiac bioenergetics.

### rRELMα Impairs Mitochondrial Ultrastructure and Affects Mitochondrial DNA Replication-Related Genes

Finally, we sought to evaluate whether the changes in cellular metabolism were also secondary to changes in mitochondrial biogenesis and ultrastructure. Using transmission electron microscopy (TEM), we found that NRCMs exposed to RELMα exhibited not only increased mitochondrial heterogeneity in size and shape, but also decreased mitochondrial number, increased mitochondrial fragmentation, and alterations in mitochondrial cristae (Figure 5A). Our data clearly suggest decrease in average mitochondrial size (Figure 5B) and density per microscopic field (Figure 5C) with RELMα stimulation as compared to control. We also found decreased mitochondrial copy number (mito-DNA/ nuc-DNA ratio) upon RELMα stimulation in cardiomyocytes as compared to control (Figure 5D). In addition to regulating fatty acid oxidation, PGC-1α orchestrates mitochondrial turnover by inducing the expression of genes required for mitochondrial biogenesis and mitochondrial DNA replication and maintenance. Quantitative PCR revealed that exposure to RELMα significantly downregulated mitochondrial transcription factor A *(TFAM)*, a key player in mitochondrial DNA replication, along with other genes encoding enzymes required for mitochondrial DNA replication such as mitochondrial topoisomerase I (*Top1mt*), mitochondrial DNA polymerase subunit gamma 2 (*POLG2*), and mitochondrial DNA-directed RNA polymerase (*Polrmt*) in NRCMs (Figure 5E). These data indicate that RELMα caused changes in mitochondrial ultrastructure, biogenesis, and its replication machinery.

## Discussion

The treatment for arterial hypertension has dramatically improved outcomes for patients in recent years. However, PAH remains a lethal disease mainly due to RV failure and therefore load-independent RV targeted therapies have been postulated as an additional modality to treat PAH. Metabolic remodeling appears to be a consistently associated with RV failure (13, 24); however, the mechanisms explaining this metabolic remodeling are still incomplete. Our study demonstrates that RELMα, a member of the family of resistin-like molecules, is sufficient to cause a significant change in cardiac metabolism *in vitro*. rRELMα produced a significant decrease in the expression of the master regulator of oxidative metabolism and mitochondrial turnover, PGC-1α, along with its corresponding transcription factors (PPARα and ERRα). To test whether the changes in protein expression translated into dysfunctional mitochondrial respiration, we tested the effect of rRELMα on isolated cardiomyocytes with specialized microplates that allowed us to measure cellular oxygen consumption and extracellular acidification rates in real time. These bioenergetic measurements confirmed that RELMα-exposed cardiac cells had decreased mitochondrial oxidative capacity, specifically when the fatty acid palmitate was used as the main energy substrate. Lastly, we showed that exposure to RELMα *in vitro* not only affected mitochondrial oxidative capacity but also mitochondrial ultrastructure.

Multiple mechanisms underlying the transition between adaptive and maladaptive cardiac hypertrophy in the right heart have been proposed in recent years (7, 25), including capillary rarefaction (6, 26), cardiac fibrosis (3, 27), and metabolic remodeling (13). Perhaps most importantly, experimental data have suggested that these mechanisms cannot be explained entirely by pressure-overload (6); therefore load-independent factors have been proposed to contribute to right heart failure (28). Metabolic remodeling has long been considered a critical pathological process in left heart failure, which is associated with abnormal mitochondrial respiration, compromised cardiac energy production, and impaired mitochondrial biogenesis (23). Similar to left heart failure, right heart failure in rats and humans appears to be characterized by metabolic gene remodeling and mitochondrial dysfunction (13), as genes related to oxidative metabolism are downregulated, and those related to glycolysis are upregulated (13, 29, 30). Despite the recent interest in the role of metabolic remodeling during heart failure; however, the stimuli that initiate or perpetuate the change in cardiac energetics remain incompletely understood. RELMα becomes highly upregulated in the lung in response to chronic hypoxia and inflammation (31, 32). We have previously shown that RELMα has proliferative, angiogenic, vasoconstrictive, and chemokine-like properties that are associated with the development of PAH (8, 12, 21, 33, 34). Furthermore, RELMα knockdown partially attenuates hypoxia-induced lung vascular remodeling and RV hypertrophy in rats, whereas RELMα gene delivery into the lungs produces PAH and RV hypertrophy (8). Lastly, mice injected with rRELMα through the tail vein exhibit increased pulmonary arterial pressures, lung vascular remodeling, and RV hypertrophy (12). Altogether, we postulate that RELMα secreted from a sick lung circulation could perhaps help explain the load-independent changes in metabolic remodeling seen in animals and patients with severe RV failure secondary to PAH. Recent study (35, 36) have demonstrated that adenovirus mediated RELMα over expression in neonatal rat cardiomyocytes can cause cardiac dysfunctions by increasing IL-6 and resting intracellular Ca^2+^ concentration and activating the CaN–NFAT (calcineurin–nuclear factor of activated T cell) and MAPK (mitogen activated protein kinase) pathways. Interestingly, we found increased RELMα/ HIMF, as well as mitochondrial metabolic gene expression in hypoxic RV tissue (Supplementary Figures 2F,G) highlighting its important role in mediating downstream hypertrophic and/or cardiometabolic dysfunctions. Generally, severe proton leak mediated by opening of mitochondrial permeability transition pore (MPTP) is detrimental to mitochondria and adversely impact the cell viability (37). This observation was explained by differential mechanisms that are responsible for promoting mild proton leak and intensive proton leak. Interestingly, our data suggest no intensive mitochondrial proton leakage in RELMα treated NRCMs (Figure 2D, right panel), but significant decrease in mitochondrial biogenesis, ultrastructure (Figure 5) and ATP-linked OCR (Figure 2D, left panel), which may stimulate NRCMs to undergo improved cell viability and size (Supplementary Figures 1A,B) as an initial adaptive hypertrophic response to compensate the dysregulated mitochondrial bioenergetics status in RELMα-treated cardiomyocytes. This might be a reason of no major difference observed in OCR-based proton leak following oligomycin injection as compared to control (Figure 2B). Our data also suggest RELMα stimulation in cardiomyocytes particularly limits FA availability for sequential mitochondrial β-oxidation (FAO), tricarboxylic acid (TCA) cycle and oxidative phosphorylation (OXPHOS) signaling pathways required for ATP production through ETC. In addition, being a pro-inflammatory molecule RELMα also induced inflammatory cytokines IL-1β, IL-6, IL-8, and NLRP3 genes (Supplementary Figure 3) widely associated with dysregulated mitochondrial bioenergetics and cardiac remodeling (38) in NRCMs. Thus, prolonged scarcity of FA (attenuate oxidative phosphorylation machinery) due to RELMα, in parallel to inflammasome complex activation (dysregulates mitochondrial ATP-linked OCR, biogenesis, and ultrastructure) might be responsible for their synergistic impact on myocardial bioenergetics, thus, cardiac dysfunction. Our study highlights RELMα mediated mitochondrial dysfunction could be an early therapeutic target for cardiovascular diseases. However, to further establish its mechanistic detail, other important components of mitochondrial metabolites (triglycerides, glycogen etc.) associated with signaling pathways like glycolysis, pentose phosphate, TCA cycle and OXPHOS should be explored deeply by using specific inhibitor and/or neutralizing antibody approach. *In vivo* elucidation of receptor mediated signaling axis like inflammation, calcium imbalance, endoplasmic reticulum (ER) stress and redox homeostasis could also be considered to unravel the mechanism(s) associated with RELMα-dysregulated mitochondrial bioenergetics in cardiomyocytes. These findings shed light on the potential mitochondrial metabolic role of RELMα not only in the development and progression of vascular remodeling in PAH but also directly on the pathobiology of RV failure. RELMα could potentially become a target to treat both pathological vascular remodeling in the lungs and metabolic remodeling in the failing heart.

## Conclusions

Our data suggest that RELMα can directly affect cardiac bioenergetics, mitochondrial metabolism, structure, function, and its biogenesis, in part by downregulating expression of the PGC-1α/PPARα/ERRα axis.

## Data Availability Statement

The raw data supporting the conclusions of this article will be made available by the authors, without undue reservation.

## Ethics Statement

The animal study was reviewed and approved by Johns Hopkins Medical Institutes Animal Care and Use Committee.

## Author Contributions

BT, SK, JG-A, CF, AZ, JS, EH, and IS has done the experiments. SK, WZ, QL, KY-K, and AH had helped in data analysis and laid the foundation of the manuscript. WG and RJ has supervised them and conceived the entire project. All authors contributed to the article and approved the submitted version.

## Conflict of Interest

The authors declare that the research was conducted in the absence of any commercial or financial relationships that could be construed as a potential conflict of interest.
