# Supplementary material for: Resistin-Like Molecule α Dysregulates Cardiac Bioenergetics in Neonatal Rat Cardiomyocytes
Source: Front Cardiovasc Med. 2021 Apr 26;8:574708. doi: 10.3389/fcvm.2021.574708 (PMC8107692; doi:10.3389/fcvm.2021.574708)
Supplement: Supplementary file 1 [file Data_Sheet_1.pdf]

Supplementary Materials

Table 1. Quantitative PCR primers

Gene	Forward sequence	Reverse sequence
PGC-1α	5′- ACCCCAGAGTCACCAAATGA-3′	5′- GCAGTTCCAGAGAGTTCCAC-3′
PPARα	5′- GGTCCTCTGGTTGTCCCCTT-3′	5′- GTGAGTTACGCCCAAATGCA-3′
ERRα	5′-CACAAGGAGGAGGAGGATGG-3′	5′-AGGCGTTTGGGTAGAGAGCT-3′
ACADS	5′-CTGGATTGTGCCGTGAAGTAC-3′	5′-GCTTGAACTGGATGTTTTGGA-3′
ACADM	5′-GACGGAGCAGCAGAAAGAGTT-3′	5′-CTTGATGAGAGGGAACGGGTA-3′
ACADL	5′-AAGTGAATGACCCTGCCAAGA-3′	5′-ATGCCCCCAGTTCTTTGAGTC-3′
CD36	5′-GTTATTGGTGCTGTCCTGGCT-3′	5′-CCGTTTTCACCCAGTTTTTGA-3′
CPT-1a	5′-GGCATGATCGCAAAGATCAGT-3′	5′-GCCACCCAGAGCCCTGTACCA-3′
GLUT4	5′-AGAGTCTAAAGCGCCT-3′	5′-CCGAGACCAACGTGAA-3′
CS	5′-GCATGGACTAGCAAACCAGG-3′	5′-ATATCCTGGGACAACCCGTC-3′
PDH1	5′-CACGGACCATCTCATCACTG-3′	5′-TAGCACAGCCTCCTCTTCGT-3′
PDK1	5′-ACAATTCACGGAATGCCCCT-3′	5′-TACTTGGCGTAGAGACGGGA-3′
PDK4	5’-CAAAGATGCTCTGCGATCAGTA-3’	5’-TGGTGAAGGTGTGAAGGAAC-3’
Hexokinase-2	5’-GCTGTGAAAATGTTGCCTACC-3’	5’-CATTGTCCGTCACCCTTACTC-3’
Mitochondrial-D loop region	5’CCTCCCATTCATTATCGCCGCCCTTGC-3’	5’GTCTGGGTCTCCTAGTAGGTCTGGGAA-3’
18S rRNA	5’-GCAATAACAGGTCTGTGATGCC-3’	5’CACGAATGGGGTTCAACG-3’
ANP	5’-GGTAGGATTGACAGGATTGGA-3’	5’-GCAGATTTGGCTGTTATCTTCG-3’
BNP	5’-CAGAACAATCCACGATGCCAGA-3’	5’-GCGCTGTCTTGAGACCTAAG-3’
β-MHC	5’-TGACCTGAAGCTGACCCA-3’	5’-CCTGCTCATCCTCAATCCTG-3’
Cpt-1b	5’-AGCAACTATTACGCCATGGATT-3’	5’-GTACCATACCCAGTGCCATC-3’
LCAD	5’-CACAAAAGAACAGATCGAGCAG-3’	5’-AGAATCCAATCACTCCCAGAC-3’
VLCAD	5’-CCTGCCAAGAATGACTCCTT-3’	5’-CACAATCTCTGCCAAGCGA-3’
RELMα	5’-GCTGGGATGACTGCTACTGG-3’	5’-AAACGGGGTTAATGGGCAAT-3’
POLG2	5’-CTGAGTAAGGAACAGCTAGTGG-3’	5’-ATCCAGGCAGTGAACATAGTG-3’
POLRMT	5’-GTGAGCAAGCTGTCCAGA-3’	5’-ACCTTCTTCACCCTCATCTCA-3’
TFAM	5’-AGCTAAACACCCAGATGCAA-3’	5’-GTACACCTTCCACTCAGCTTT-3’
TOP1MT	5’CTGCATCTCGAGTGTACGAC-3’	5’-GTGTGTGATGAGCTTCCTCTC-3’
IL-1β	5’-GTGCTGTCTGACCCATGT-3’	5’-TTGTCGTTGCTTGTCTCTCC-3’
IL-6	5’-CAGAGCAATACTGAAACCCTAGT-3’	5’-CCTTCTGTGACTCTAACTTCTCC-3’
IL-18	5’-CGGAGCATAAATGACCAAGTTC-3’	5’-GCCAGTCCTCTTACTTACTTCACTATC-3’
NLRP3	5’-TTTCCCAGACCCTCATGTTG-3’	5’-GTGCTGAGACTTGAGAAGAGAC-3’

Supplemental Figures:

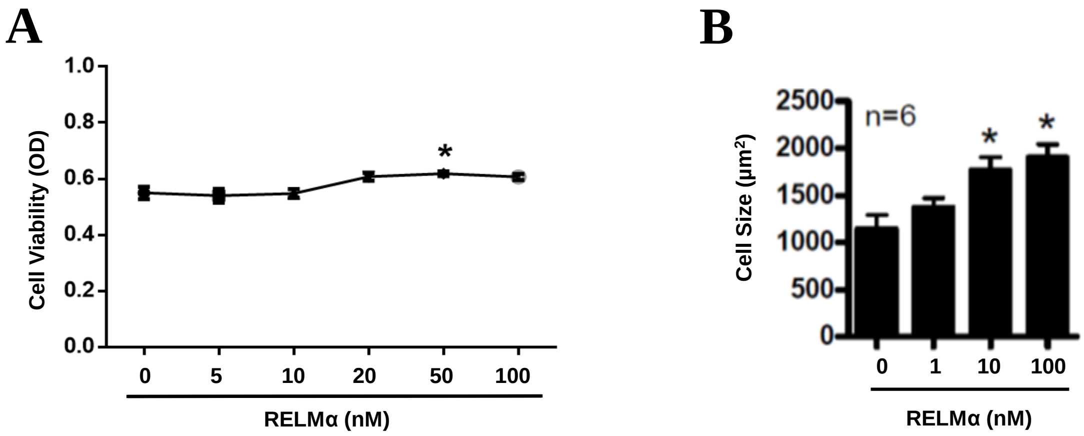

Figure S1: Effects of rRELMα on cardiomyocytes viability and size. (A) Cell viability evaluated by colorimetric BrdU assay in neonatal rat cardiomyocytes stimulated with 0 to 100 nM RELMα. n=6/group. (B) Quantification of the cell surface area with indicated doses of RELMα in NRCMs. n=50-100 cells per group and n= 6/group. **p*<0.05 vs without RELMα group.

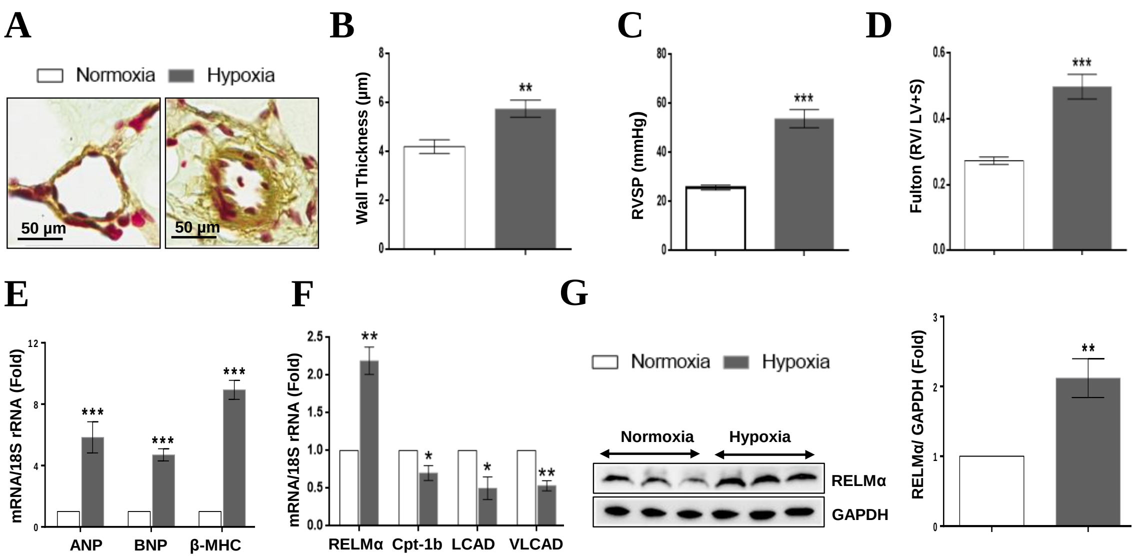

Figure S2: Effects of hypoxia-induced pulmonary hypertension and right ventricular hypertrophy on RELMα and mitochondrial metabolic genes. ***(A, B)*** Representative images and its relative quantification from rat lung tissue sections stained for VWF (von Willebrand factor; brown) and α-SMA (α-smooth muscle actin; red) to define the medial vessel wall thickness. n= 100-150 medial vessel count per group and N= 6 rats/group**.** *(C, D)* Hemodynamic analysis, right ventricular systolic pressure (RVSP), and Fulton index [right ventricle (RV) weight/ left ventricle (LV) + septum (S) weight] from rats RV tissue. N= 6 rats/group**.** *(E F)* Quantitative PCR analyses of atrial natriuretic peptide (ANP), brain natriuretic peptide (BNP), beta-myosin heavy chain (β-MHC), RELMα (Resistin like molecule α), carnitine palmitoyltransferase-1b (CPT-1b), long-chain acyl-CoA dehydrogenase (LCAD), and very long-chain acyl-CoA dehydrogenase (VLCAD) in total mRNA derived from rats RV tissue. N=3-4 rats/group. *(G)* Immunoblotting (left) and statistical (right) analysis RELMα in RV tissue of indicated rat group. N= 6 rats/group. *, **, ***p<0.05, 0.01, 0.001 vs. Normoxia.

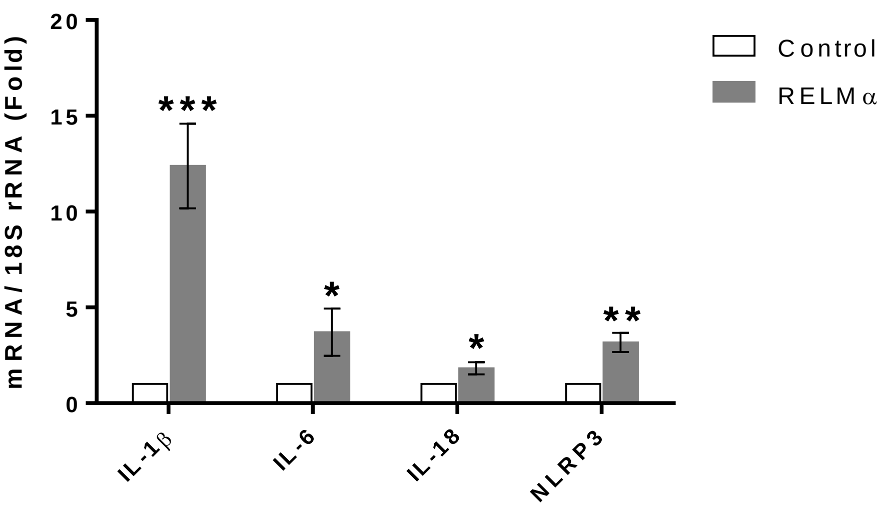

Figure S3: Effects of rRELMα on inflammatory cytokines and NLRP3 in NRCMs. qPCR analyses of cytokines (IL-1β, IL-6, IL-18) and NLRP3 expression from total mRNA derived from NRCMs group treated with PBS or rRELMα (50 nM, 24h). n= 5/group. *,****p*<0.05, 0.001 vs. control.

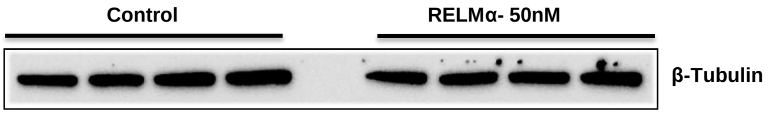

Figure S4: Representative immunoblotting of beta-Tubulin with or without RELMα (50 nM) stimulation in NRCMs. n=4/group.

Sea Horse Image (Figure 4A) Approval from Agilent Technologies, Inc.

Agilent

Agilent Technologies, Inc. Intellectual Property Group
5301 Stevens Creek Blvd., MS1A-PB
Santa Clara, California 95051

Janet Shih Hajek Corporate Counsel

303 588 7502 telephone
janet.hajek@agilent.com
www.agilent.com

April 21, 2020

Via e-mail: skumar46@jhmi.edu; santoshszu1517@outlook.com

Santosh Kumar, Ph.D.
Johns Hopkins Medical Institutions
720 Rutland Ave, Ross 361
Baltimore, MD 21205

Re: Permission to Copy and Republish Agilent Image

Dear Dr. Kumar:

This is in response to your request seeking consent to copy, modify and republish an image in which Agilent Technologies, Inc. ("Agilent") owns the copyright. Specifically, you have requested to copy and modify the image shown below (the "Agilent Image") to be republished in a manuscript that you are authoring entitled, "Resistin-like Molecule Alpha Dysregulates Cardiac Bioenergetics in Neonatal Rat Cardiomyocytes" to be submitted to the journal of ***Frontier in Pharmacology*** (the "Work").

Agilent Image:

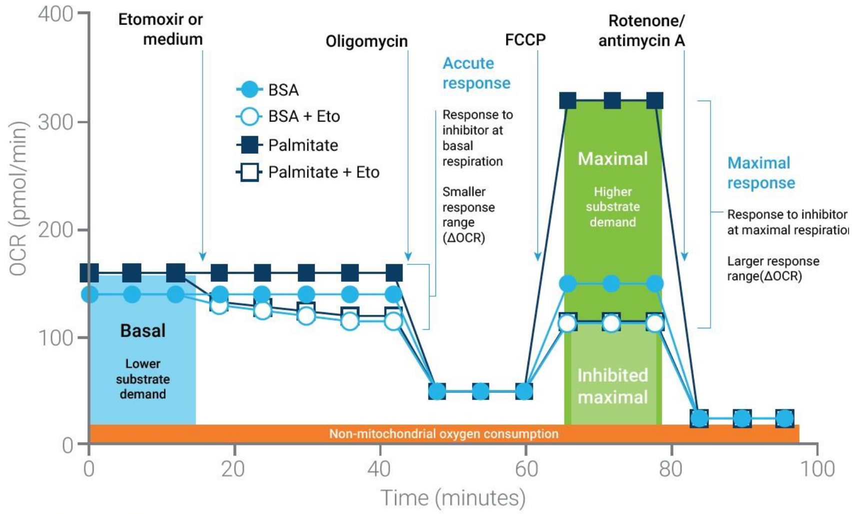

Figure 2. Advanced assay output

See: https://www.agilent.com/cs/library/usermanuals/public/user-manual-substrate-oxidation- stress-test-kits-cell-analysis-5994-1164en-agilent.pdf (page 7).

Agilent

I am pleased to advise that you have Agilent's permission to copy, modify and republish the Agilent Image in the Work. This consent extends to the Agilent Image as used in the Work, and all subsequent editions and derivative works based on the Work, in any and all media of expression now known or later developed, and in all languages throughout the world.

This consent is conditioned upon there being included with the Agilent Image an appropriate acknowledgement and copyright notice in a form similar to the following:

© Agilent Technologies, Inc. 2020
Reproduced with Permission, Courtesy of Agilent Technologies, Inc.

By modifying and reproducing the Agilent Image, you acknowledge and agree: (1) that the Agilent Image and the information contained therein are accepted "AS IS"; (2) that Agilent does not make any representations or warranties of any kind with respect to the Agilent Image and disclaims any responsibility for the content as reproduced by you; (3) that Agilent reserves the right to review any use of the Agilent Image and to terminate this consent and require the Agilent Image's immediate removal if altered, modified, or portrayed in any way that, in Agilent's opinion, is in poor taste or diminishes the reputation of Agilent or its products; (4) that Agilent will not be liable for any damages, whether special, incidental, consequential, direct, indirect or punitive, resulting from or in connection with the use, copying or disclosure of the Agilent Image, and (5) to indemnify Agilent for all claims,
actions, damages, and expenses (including attorneys' fees) incurred by Agilent as a result of the Agilent Image being reproduced, modified, or distributed pursuant to this consent.

This limited consent does not grant a license to use any Agilent trademark. Except for the limited consent to use the Agilent Image referenced herein, this limited consent does not grant a license to use any other Agilent copyrighted work.

Thank you for your interest in Agilent and its products and services.

Sincerely,

Janet Shih Hajek Corporate
Counsel Agilent
Technologies, Inc.
